# Genomic Sequence of *Burkholderia multivorans* NKI379, a Soil Bacterium That Inhibits the Growth of *Burkholderia pseudomallei*

**DOI:** 10.1128/genomeA.01294-15

**Published:** 2015-11-19

**Authors:** Pei-Tan Hsueh, Jong-Kang Liu, Ya-Lei Chen, Pei-Ju Liu, Wen-Fan Ni, Yao-Shen Chen, Keh-Ming Wu, Hsi-Hsun Lin

**Affiliations:** a Department of Biological Science, National Sun Yat-sen University, Kaohsiung, Taiwan; b Department of Biotechnology, National Kaohsiung Normal University, Kaohsiung, Taiwan; c Department of Internal Medicine, Kaohsiung Veterans General Hospital, Kaohsiung, Taiwan/Department of Internal Medicine, National Yang-Ming University, Taipei, Taiwan; d Welgene Biotech, Inc., Taipei, Taiwan; e Department of Medicine, Section of Infectious Disease, E-Da Hospital, Kaohsiung City, Taiwan

## Abstract

*Burkholderia multivorans* NKI379 is a soil bacterium that exhibits an antagonistic effect against the growth of *Burkholderia pseudomallei*, the causative agent of the infectious disease melioidosis. We report the draft genomic sequence of *B. multivorans* NKI379, which has a G+C content of 67% and 5,203 candidate protein-encoding genes.

## GENOME ANNOUNCEMENT

*Burkholderia multivorans*, a member of the *Burkholderia cepacia* complex, is an agent that can infect immunocompromised patients and those with cystic fibrosis (1). However, *B. multivorans* strains have been used in attempts to develop insecticidal or miticidal agents and have been used as fertilizer supplements to improve plant growth (2, 3). *B. multivorans* strain NKI379 was first isolated from fertilized agricultural soil in Taiwan (4). This bacterium exhibited an antagonistic effect against the growth of *Burkholderia pseudomallei*, a soil-borne infectious agent that causes life-threatening melioidosis in humans and animals in Southeast Asia and northern Australia (4, 5). To develop *B. multivorans* NKI379 as a biological control agent against melioidosis in agriculture, the potentially virulent and antimicrobial genes of this bacterium were sequenced using next-generation sequencing technology.

Total DNA was extracted from *B. multivorans* NKI379 using a WelPrep DNA kit (Welgene Biotech, Taiwan) according to the manufacturer’s instructions. The genomic DNA was sequenced using an Illumina MiSeq sequencer (Illumina Inc., San Diego, CA, USA). The DNA end-repair, A-tailing, and adaptor-ligation protocols of the TruSeq kit were followed per the manufacturer’s instructions (Illumina). In this study, a short-insert library with an average insert size of 450 bp was sequenced, generating 13,241,890 pass-filter reads totaling 3,972,567,000 bp of Illumina data. Unclear reads were trimmed or removed using Trimmomatic (version 0.32) with a quality threshold of Q20 (>99% accuracy) (6). The trimmed reads were *de novo* assembled using Velvet (version 1.2.09), and the gene annotation was performed using MAKER pipeline (version 2.28) (7, 8).

The genomic sequence of *B. multivorans* NKI379 was found to contain a G+C content of 67% and 5,203 candidate protein-encoding genes. A total of 9 protease genes (YP_001946424, WP_006413365, YP_001948758, WP_006396933, YP_001580205, YP_001580356, YP_003709042, WP_006407220, and YP_00194878), 4 hemolysin genes (WP_006400323, WP_006414151, YP_001578869, and WP_006413720) and a burkholderial toxic protein (WP_006404794) with potential roles in bacterial virulence were identified. Two antimicrobial gene sequences encoding linocin M18 (P_001585375) and colicin V (YP_001584586) as well as a putative bacteriocin secretion protein gene sequence (WP_006399594) were also identified.

### Nucleotide sequence accession number.

The whole-genome sequence of *B. multivorans* NKI379 has been deposited at GenBank under the accession number LJJG00000000.
